# Chemical composition and fatty acid content in lamb and adult sheep meat

**DOI:** 10.5194/aab-63-261-2020

**Published:** 2020-07-24

**Authors:** Andrzej Junkuszew, Paulina Nazar, Michał Milerski, Milan Margetin, Piotr Brodzki, Konrad Bazewicz

**Affiliations:** 1 Faculty of Animal Sciences and Bioeconomy, Institute of Animal Breeding and Biodiversity Conservation, University of Life Sciences in Lublin, Lublin, Poland; 2 Department of Genetics and Breeding of Farm Animals, Institute of Animal Science, Prague, Czech Republic; 3 Department of Animal Production, Faculty of Agrobiology and Food Resources, Slovak University of Agriculture in Nitra, Nitra, Slovak Republic; 4 National Agricultural and Food Centre, Research Institute for Animal Production Nitra, Nitra, Slovak Republic; 5 Department of Andrology and Biotechnology of Animal Reproduction, University of Life Sciences in Lublin, Lublin, Poland

## Abstract

The aim of the present study was to compare the chemical composition and fatty acid (FA) content of the muscle tissues of lambs and adult sheep bred for meat production. Throughout the research period, the animals were managed in a confinement indoor system under uniform environmental conditions. After slaughter, meat samples collected from the *musculus biceps femoris* were used to determine the basic chemical composition and fatty acid content in the intramuscular fat. The analyses showed that, compared to lambs, meat from adult animals exhibited a more beneficial ratio of n-6 to n-3 FAs, as well as a favorable percentage of OFAs (dietary fatty acids having an undesirable hypercholesterolemic effect on humans). The results of this study support the inclusion of dietitian-recommended mutton into the human diet and the promotion of the production of meat from adult animals as a high-value product. The sheep industry could benefit economically, particularly in countries where this meat type has not enjoyed a high standing in consumer preferences.

## 1 Introduction

Diet-related diseases are considered one of the leading causes of mortality in western European countries and the USA. These diseases are directly linked to the advancing development of contemporary civilization, and, as hazard scales indicate, they afflict approximately 50 %–60 % of the adult population in these countries. Notably, diet-related diseases are generally nonexistent or rare in hunter–gatherer societies, in which the nutritional patterns differ markedly from those of westernized cultures (Cordain et al., 2005). Increasing societal wealth is a major determinant of increased meat consumption and associated meat production (Speedy, 2003). Kourlaba and Panagiotakos (2009) thus suggest that research should focus on the development of dietary guidelines centered on the primary prevention of lifestyle-related diseases that can impose a substantial burden on society.

One of the major factors associated with increased risk of lifestyle-related diseases is a high intake of saturated fatty acids (SFAs), which can increase levels of blood cholesterol and, as has been well established, contribute to atherosclerotic growth. A report by the FAO/WHO concluded that dietary replacement of SFAs with polyunsaturated fatty acids (PUFAs) decreases the risk of coronary heart disease (FAO, 2010). According to the recommendations of dietitians and physicians, meat can be considered a healthy food if it is low in fat with an appropriate fatty acid (FA) profile. It is vital that both n-3 and n-6 FAs are present in meat at sufficient levels. However, research has shown that the n-6 / n-3 FA ratio is 15/1 to 16.7/1 in the typical western diet, leading to increased incidences of a number of lifestyle-related diseases. Importantly, a lower n-6 / n-3 FA ratio can reduce the risk of these diseases (Simopoulos, 2008), particularly cardiovascular disease (Nestel et al., 2015; Wen et al., 2014; Endo and Arita, 2016), cancer, and respiratory diseases (Simopoulos, 2008; Gold et al., 2016; Rovito et al., 2015; Wiggins et al., 2015). FAs play critical roles in immune response development and the transport of fat-soluble vitamins such as vitamins D, E, and K (Webb and O'Neill, 2008). Although the vast majority of scientific papers published to date have focused on PUFAs, the role of other FAs should not be neglected. Recent reports have highlighted the vital function of stearic acid in lowering the low-density lipoprotein (LDL) cholesterol level, whereas until very recently this FA was considered a thrombosis-promoting agent (Li et al., 2005; Hunter, 2010).

As meat production and consumption, including that of sheep meat, is projected to continue growing (Speedy, 2003; OECD, 2015), special attention should be paid to livestock product quality. It is necessary to determine the nutritive value of each type of meat and identify the factors affecting its chemical composition and, consequently, the quality of the consumed food. Sheep are widely recognized as a good source of valuable, high-quality meat. Research has confirmed that sheep meat is rich in many vitamins, minerals, and essential PUFAs (Ponnampalam et al., 2016b). Nevertheless, in the case of sheep, mutton (meat of adult animals) is frequently underrated as a culinary product because of stereotypes associated with it. Consumer unwillingness to consume mutton is likely due to its excessive fattiness, which is frequently associated with inferior sensory attributes. Pethick et al. (2005) reported similar findings that confirmed the higher palatability of lamb. However, they stated that the aversion of customers to so-called “mutton flavor” is virtually insignificant if the content of back fat and intramuscular fat (marbling) is reduced. According to the present authors, this undervalued meat deserves far more attention, especially considering the marked and significant reductions in animal fattiness in recent years and the increasing importance of the production of sheep as meat sources. Obviously, selective breeding of sheep for meat production has affected the chemical composition of the obtained meat. Taking into account the fact that product quality is rapidly becoming a chief consumer concern, comparative studies are needed that focus on the chemical composition and profile of FA content in the muscle tissues of lambs and adult sheep bred for meat production. The research results can be helpful for promoting the consumption of meat from adult animals as a high-value product, which should consequently improve the profitability of sheep production.

The aim of the present study was to compare the chemical composition and fatty acid content of the muscle tissues of lambs and adult sheep bred for meat production.

## 2 Materials and methods

The study was conducted at Bezek research station, a part of the University of Life Sciences in Lublin, located in southeastern Poland. The farm houses 550 ewes in an indoor pasture system. Lambing occurred in January, and the lambs remained with their ewes in the sheep house throughout the rearing period.

The experiment was carried out in such a way that normal daily activities on the farm were not disturbed, but it was ensured that all the conditions for the care of the animals were in line with the standards recommended in the Guide for the Care and Use of Laboratory Animals, Directive 2010/63/EU and Directive 1998/58/EU. This study did not require ethical consent because all the procedures were breeding procedures. The study was carried out when selling animals to the slaughterhouse, which is a standard procedure in animal breeding. Samples constituting the commercial product of a commercial slaughterhouse were bought for chemical analysis. At the same time, the authors declare that all animals kept in the herd were under the constant control of the advisory team for animal welfare of the Faculty of Animal Breeding and Bioeconomy of the University of Life Sciences in Lublin.

### 2.1 Animal material

The study involved 30 animals of the synthetic prolific meat line SCP (15 adult ewes and 15 lambs). The synthetic prolific meat line is composed of the following sheep breeds: Polish Lowland, 37.5 %; Romanowska, 12.5 %; Suffolk, 25.0 %; and Charolaise, 25.0 %. Throughout the research period, the animals were managed in the confinement housing system (indoors) under uniform environmental conditions. Both ewes and their progeny were housed in the same flock and building from birth until slaughter. During the experiment, the animals were fed feed available at the farm in the doses presented in Table 1. All of the lambs included in the study came from twin births and remained with the ewes over the entire study period.

**Table 1:** Approximate feed ration used for ewes and offspring (kg).

	January		February		March		April	
	Ewes	Lambs	Ewes	Lambs	Ewes	Lambs	Ewes	Lambs
Mother's milk		ad libitum		ad libitum		ad libitum		ad libitum
Crushed oats	0.4		0.5	0.03	0.5	0.2	0.5	0.25
Bran				0.03		0.2		0.25
Dried sugar-beet pulp				0.02		0.05		0.05
Rape seed meal extract	0.05		0.05		0.05		0.05	
Soybean meal						0.02		0.02
Hay	0.8		0.8	0.1	0.8	0.2	0.8	0.2
Green silage	2.5		2.5		2.5	0.8	2.5	0.8

### 2.2 Evaluation of slaughtered animals

After the lamb rearing period, both ewes and their offspring were slaughtered. The age at slaughter was similar in both groups (i.e., lambs 100±4 d and ewes 6 years; for the past 20 years, lambing occurred in January at the research station, so the difference in ewe age was 24 d). Animal slaughter was performed on the same day.

The resulting animal carcasses were evaluated according to the SEUROP scale (S – 6, E – 5, U – 4, R – 3, O – 2, P – 1) and then chilled at 4 ${}^{\circ }$C for 24 h.

### 2.3 Chemical analysis of muscle tissue

Meat samples collected from the *musculus biceps femoris* (MBF) were examined to determine the basic chemical composition and FA profile of the intramuscular fat. All chemical determinations were carried out in the approved laboratory.

### 2.4 Analysis of basic chemical composition of muscle and determination of caloric value

Each sample (approximately 250 g in weight) was placed in a sterile, tightly closed Ziplock-type bag and stored under the same cooling conditions. The analysis of the basic chemical composition (water, dry matter, fat, and collagen content) was performed using near-infrared (NIR) spectroscopy. The meat specimens (200 g) were homogenized with a cutter mixer (model K 35; DITO Electrolux) and transferred to a cuvette FoodScan™ analyzer operated in NIR transmission mode in the region of 850–1050 nm and equipped with a calibration artificial neural network (model developed using artificial neural networks) according to norm PN-A-82109:2010.

On the basis of the results obtained with norm PN-A-79011-6:1998, meat caloric values were calculated using Atwater factors. Total ash content was established using a gravimetric method according to norm PN-ISO 936:2000 including drying, charring, and final incineration at 550±25 ${}^{\circ }$C. After cooling, the residue mass was determined.

### 2.5 Analysis of FA content

The FA profile was established using the intramuscular fat. FA methyl esters were determined according to the guidelines specified in PN-ISO 5509:1996, whereas FA composition was determined by gas chromatography using a Hewlett-Packard 6890 (Agilent Technologies) instrument equipped with a flame ionization detector and a highly polarized BPX70 column. The column used was 60 m × 0.25 mm i.d. ×0.25 µm film thickness. The BPX70 column oven program was as follows: 100 ${}^{\circ }$C for 0.5 min, temperature program at 20 ${}^{\circ }$C min${}^{-1}$ to 130 ${}^{\circ }$C, hold isothermally for 2 min, temperature program at 1 ${}^{\circ }$C min${}^{-1}$ to 150 ${}^{\circ }$C, hold isothermally for 3 min, temperature program at 3 ${}^{\circ }$C min${}^{-1}$ to 220 ${}^{\circ }$C, and hold isothermally for 6 min.

FAs were identified by the comparison of retention times with those of the standard mixture FAME (Supelco 37 Component FAME Mix and C18 FAME isomers; Sigma-Aldrich Co.).

### 2.6 Statistical analysis

The results were analyzed statistically using the one-way multivariate analysis of variance (ANOVA) and the Statistica data analysis software system version 13 (Dell Inc., 2016). The experimental factor analyzed was the type of meat (i.e., mutton, lamb).

## 3 Results

The assessment results of dressing percentage and lamb carcass conformation and fatness using the SEUROP classification are important for potential customers (Table 2). Dressing percentage of adult sheep was 3.69 percentage points higher than that of lambs, whereas SEUROP classification showed a 0.2 point higher lamb score compared to adult carcasses. In the case of fatness, the tendency was opposite. Carcasses of adult sheep obtained a higher fatness rating by 0.06 points.

**Table 2:** Characteristics of slaughter material.

	Ewes	Lambs
	Mean ± SD	Mean ± SD
Body weight (kg)	72.33 ± 10.33	29.00 ± 5.56
Carcass weight (kg)	32.35 ± 6.56	12.07 ± 2.65
Dressing percentage	45.10 ± 9.18	41.41 ± 1.68
SEUROP meat	3.47 ± 0.81	3.67 ± 1.19
SEUROP fat	3.13 ± 0.72	3.07 ± 0.77

S – 6, E – 5, U – 4, R – 3, O – 2, P – 1.

The chemical composition of food products significantly affects their quality. The results from the analysis of the basic chemical composition of the meat of mature ewes and lambs are presented in Table 3. A higher fat content in the MBF was noted in adult sheep. The intramuscular fat percentage determined was 1.5 times higher in the mature animal muscle tissues compared to lamb meat (P≤0.01). However, an inverse relationship was observed for the percent of protein content in the analyzed muscle. The values obtained for adult ewes were at 18.96 % versus 20.73 % for lamb meat (P≤0.001). The analysis of meat caloric value did not show any statistically significant difference between lamb meat and mutton.

**Table 3:** Quality traits of ewe and lamb *musculus biceps femoris* meat.

	Ewes	Lambs	Significance
	Mean ± SD	Mean ± SD	
Fat (%)	5.46 ± 1.76	3.69 ± 0.94	${}^{**}$
Protein (%)	18.96 ± 0.45	20.73 ± 0.32	${}^{***}$
Moisture content (%)	72.69 ± 2.90	75.11 ± 0.93	${}^{**}$
Collagen (%)	1.34 ± 0.18	1.31 ± 0.21	ns
Ash (%)	1.07 ± 0.03	1.11 ± 0.04	${}^{**}$
Caloric value of meat (kcal)	130.36 ± 16.83	121.37 ± 9.33	ns

${}^{*}$ ≤0.05. ${}^{**}$ ≤0.01. ${}^{***}$ ≤0.001. ns: not significant.

The level of human dietary FA intake is considered critical; therefore, the amount of each FA and their group (Tables 4 and 5) were determined using 100 g of meat (mg per 100 g of meat). In almost all cases, a higher FA content in the MBF was noted in adult sheep. Exceptions were acids from C${}_{10:0}$ to C${}_{14:0}$ and C${}_{20:3}$ n-6 (Table 4). It is worth noting that the muscles of sheep mothers showed an approximately 1.4 times higher content of C${}_{16:0}$ FA compared to their progeny (P≤0.001). The same significant difference was observed for C${}_{18:3}$ n-6 and C${}_{18:3}$ n-3 acids. In the case of C${}_{18:3}$ n-6 acids, the values obtained for adult ewes were 9.98 mg per 100 g of meat versus 5.15 mg per 100 g of meat for lambs. The C${}_{18:3}$ n-3 FA content was 40.3 mg per 100 g of meat and 15.5 mg per 100 g of meat in ewes and lambs, respectively.

**Table 4:** Fatty acid selected content (mg per 100 g muscle) of ewe and lamb *musculus biceps femoris* meat.

Fatty acid	Ewes	Lambs	Significance
	Mean ± SD	Mean ± SD	
C${}_{10:0}$	6.11 ± 2.18	13.44 ± 4.88	${}^{***}$
C${}_{12:0}$	5.48 ± 1.52	35.96 ± 12.90	${}^{***}$
C${}_{14:0}$	129.67 ± 40.98	306.26 ± 112.98	${}^{***}$
C${}_{14:1}$	8.58 ± 3.62	2.03 ± 0.64	${}^{***}$
C${}_{15:0}$	34.21 ± 12.89	29.55 ± 8.99	ns
C${}_{15:1}$	13.37 ± 4.87	11.91 ± 3.21	ns
C${}_{16:0}$	1497.84 ± 503.86	1057.84 ± 339.12	${}^{**}$
C${}_{16:1}$	140.69 ± 52.73	131.00 ± 41.49	ns
C${}_{17:0}$	80.82 ± 30.98	45.35 ± 11.28	${}^{***}$
C${}_{17:1}$	51.68 ± 20.05	34.40 ± 8.73	${}^{**}$
C${}_{18:0}$	860.90 ± 284.74	479.37 ± 104.90	${}^{***}$
C${}_{18:1}$	2397.94 ± 781.03	1369.86 ± 300.38	${}^{***}$
C${}_{18:2}$	156.46 ± 55.99	125.85 ± 26.33	ns
C${}_{18:3n6}$	9.98 ± 3.81	5.15 ± 1.52	${}^{***}$
C${}_{18:3n3}$	40.30 ± 19.16	15.50 ± 4.72	${}^{***}$
C${}_{20:0}$	5.45 ± 1.71	4.04 ± 1.13	${}^{*}$
C${}_{20:3n6}$	18.81 ± 10.41	21.95 ± 12.35	ns
C${}_{24:0}$	3.13 ± 1.00	3.06 ± 1.45	ns

${}^{*}$ ≤0.05. ${}^{**}$ ≤0.01. ${}^{***}$ ≤0.001. ns: not significant.

The content of nearly all of the acid groups differed significantly between ewes and their offspring with the lone exception being OFAs (dietary fatty acids having an undesirable hypercholesterolemic effect on humans) (Table 5).

**Table 5:** Fatty acid group content (mg per 100 g muscle) of ewe and lamb *musculus biceps femoris* meat.

Fatty acid	Ewes	Lambs	Significance
	Mean ± SD	Mean ± SD	
SFA	2621.15 ± 846.09	1977.16 ± 572.77	${}^{*}$
UFA	2837.54 ± 926.30	1715.97 ± 379.51	${}^{***}$
MUFA	2612.02 ± 854.96	1547.11 ± 350.85	${}^{***}$
PUFA	225.52 ± 78.43	168.86 ± 38.92	${}^{*}$
n-3	40.30 ± 19.16	15.50 ± 4.72	${}^{***}$
n-6	166.35 ± 58.93	130.79 ± 27.61	${}^{*}$
OFA	1627.52 ± 542.71	1364.09 ± 451.03	ns
DFA	3698.44 ± 1175.93	2195.34 ± 474.88	${}^{***}$

${}^{*}$ ≤0.05. ${}^{**}$ ≤0.01. ${}^{***}$ ≤0.001. ns: not significant. SFA: sum of all saturated fatty acids; MUFA: sum of all monounsaturated fatty acids; UFA = PUFA + MUFA; OFA: dietary fatty acids having an undesirable hypercholesterolemic effect on humans (C${}_{14:0}$ + C${}_{16:0}$); DFA: dietary fatty acids having a desirable neutral hypocholesterolemic effect on humans (UFA + C${}_{18:0}$).

**Figure 1:**
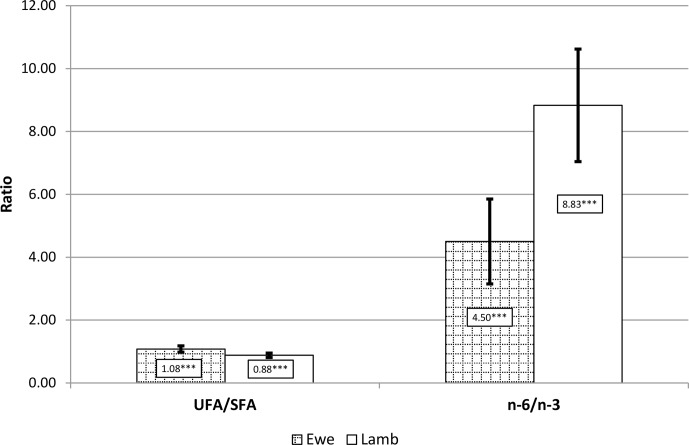
UFA / SFA and n-6 / n-3 FA ratios of ewe and lamb *musculus biceps femoris* meat (mean ± SD). The asterisks indicate significance values ≤0.001.

The dietetic value of meat depends to a great extent on the ratio UFA / SFA (UFA: unsaturated fatty acid), as well as that between n-6 FAs and n-3 FAs (Fig. 1). A higher UFA / SFA ratio was observed in mutton compared to lamb, whereas the n-6 FA / n-3 FA ratio was almost 2-fold higher in lambs. In both cases, the differences were statistically significant (P≤0.001).

## 4 Discussion

Diet is known to have a significant impact on human health. One current concern relating to proper nutrition is the rise in the prevalence of obesity as a consequence of excess caloric intake coupled with a lack of caloric expenditure. The decline in caloric expenditure is the result of decreased physical activity due to the widespread use of vehicles for transportation, work automation, and a sedentary lifestyle (Behzad et al., 2013). Considering the development of dietetic guidelines for different population groups, one should keep in mind that fat also plays a major role in proper nutrition, and FAs largely determine the health-promoting properties of meat (Fisher et al., 2000). It is also worth noting that the content of linolenic acid (LA) and alpha-linolenic acid (ALA) is a factor that limits its nutritive value because, as discussed previously, humans lacks the enzymatic capacity to synthesize LA n-6 FA and ALA n-3. In humans, LA and ALA compete with each other for metabolism by the same enzyme, delta-6 desaturase. It is important for human health as an LA intake that is too high decreases the amount of delta-6 desaturase available for ALA metabolism, which increases the risk of cardiovascular diseases and especially atherosclerosis.

A lower n-6 / n-3 FA ratio is desirable as a means of reducing the risk of a number of chronic diseases of high prevalence in both western societies and developing countries. As the present research has clearly demonstrated, the n-6 / n-3 FA ratio is markedly more beneficial in the meat of mature sheep as it was almost 2-fold higher compared to lamb's meat. Simopoulos (2008) reported that an elevated n-3 FA level has a suppressive effect on many diseases (e.g., a ratio of 4/1 is associated with a 70 % decrease in total mortality due to cardiovascular diseases, whereas a ratio of 2.5/1 reduces rectal cell proliferation in patients with colorectal cancer). Similarly, a lower dietary n-6 / n-3 ratio has been linked to a decreased risk of breast cancer in women. Notably, the ratio of these FAs determined in mutton was in compliance with dietary guidelines recommended by dietitians. Therefore, it is advisable to prioritize this information while promoting and marketing this type of meat. Benefits of a good n-6 / n-3 FA ratio in sheep fat were highlighted by Peng et al. (2010), who determined the FA content in back fat, perineal, and tail-fat tissues. Although they obtained lower ratios, this result could be attributed to the nutritional strategy applied. The n-6 / n-3 FA ratio established in the meat from adult animals in the present study was slightly lower than that determined for wild animal meat. Importantly, the meat from free-range animals is frequently recommended by doctors and dietitians with regard to a beneficial FA content. While analyzing the research results obtained in the present study, it should be noted that similar findings pertaining to the ratio of n-6 to n-3 FAs were reported for the meat of roe deer hunted in Germany. Like mutton, the FA ratio was more favorable in older animals and ranged between 2.4 and 2.6 versus 2.4 to 4.3 in juveniles (Dannenberger et al., 2013). Research indicates that the ratio of n6 to n3 FAs depends mainly on nutrition. Several studies have reported ratios in the range of 1.85 to 11.4 (Santos-Silva et al., 2002; Jerónimo et al., 2009; Ponnampalam et al., 2009; Abuelfatah et al., 2014). The reason for the higher ratio in lambs in this experiment is the use of soybean meal in feeding. Mele et al. (2007) reported that soybean meal has a significant impact on the n-6 / n-3 ratio. In farmed roe deer, in contrast, the n-6 / n-3 ratio is very close to that determined in the present research on sheep meat (Phillip et al., 2007).

Another argument in favor of promoting the consumption of meat from adult animals as a health-promoting food is the beneficial profile of OFAs (C${}_{14:0}+$ C${}_{16:0}$). Admittedly, the analysis of OFA content (mg per 100 g meat) in the present study revealed higher levels in the MBF of adult ewes, but the calculated differences were not significant. Therefore, a lower fat content in the muscle tissue should help improve the values discussed. It is worth noting that mutton was found to have both a higher stearic acid (C${}_{18:0}$) profile and muscle content, which seems beneficial in light of studies confirming the role of this acid in reducing LDL cholesterol levels and decreasing coagulation factor VII activity (Li et al., 2005; Hunter, 2010).

The present study found significant differences between the profiles and contents of particular FAs in the meat from adult ewes and lambs. These differences appear to be markedly affected by animal age and dietary factors, which, despite the fact that the animals were housed under the same conditions, must have exerted a substantial influence as the lambs were nursed by the ewes until 100 d of age. The influence of the aforementioned factors on FA profile and content has been confirmed in many other studies (Watkins et al., 2010; D'Alessandro et al., 2015; Ponnampalam et al., 2016a).

One of the objectives of the present research was to determine the dietetic value of sheep meat. It should be noted that in contrast to the meat from mature sheep, the dietetic value of lamb meat has received substantial attention in the research literature and in studies conducted recently (Sun et al., 2015; Janiszewski et al., 2016; Oliveira et al., 2016). Contrary to lamb meat, only a few scientific studies examining the meat from adult sheep have been published, and, according to the authors of those studies, this meat is of high dietetic and culinary value. In a number of countries, the meat from adult sheep is regarded as being fully equal to any other meat and is used widely (Peng et al., 2010; Ponnampalam et al., 2016a).

The present research found a higher fat content in ewe meat compared to lamb meat. The crucial finding of this study is the lack of statistically significant differences between the caloric content of the meat of mature sheep and lambs. The higher caloric value of mutton resulted primarily from the higher fat level in the MBF. The lack of confirmed significant differences along with the calculated standard deviations indicate the differentiation of this trait between the two groups. Noteworthy are the results of the SEUROP assessment, which indicate moderate carcass fat content assessed during the SEUROP classification in both lambs and adult sheep. This is especially relevant for adult sheep whose excessive fattiness is likely to be the primary reason for consumers' unwillingness to consume it (Johansen et al., 2006). It is of primary importance in the ongoing promotion of lamb meat especially since, as Bock and Connely (2008) report, consumers will willingly pay more for higher-quality premium products.

Meat market preferences for adult animal meat may be linked to price, which is usually lower compared to the meat of younger animals. As Font-i-Furnols et al. (2011) reported, consumers consider meat price the major factor affecting purchase decision-making and not, as might be expected, the feeding and housing system used. Interestingly, meat price has a greater effect when considering the buying behavior of men versus women. The desirable dietetic qualities of mutton confirmed in the present study, coupled with reasonable mutton price (which is lower than that of lamb), could contribute to a change in consumer perception (in Europe) of mutton as a meat of inferior quality.

## 5 Conclusions

The meat from adult animals exhibits a more beneficial ratio of n-6 FA to n-3 FAs compared to lamb meat, as well as a favorable percentage of OFAs (dietary fatty acids with undesirable hypercholesterolemic effects on humans). The results reported here should serve as the basis for the inclusion of dietitian-recommended mutton in the human diet and consequently should promote the consumption of meat from adult animals as a high-value product. Finally, the economics of the sheep industry could improve, particularly in countries where this meat type has not enjoyed a high standing in consumer preferences.

## Data Availability

The original data are available upon request to the corresponding author.
